# Investigation of hydrolysis of olmesartan medoxomil in different pH buffers by simultaneously measuring olmesartan medoxomil and olmesartan

**DOI:** 10.1371/journal.pone.0321142

**Published:** 2025-05-02

**Authors:** Lauren Landry, Xiaowei Dong

**Affiliations:** Department of Pharmaceutical Sciences, University of North Texas Health Science Center, Fort Worth, Texas, United States of America; Universitätsklinikum Magdeburg: Universitatsklinikum Magdeburg, GERMANY

## Abstract

Olmesartan medoxomil (OLM) is an ester prodrug of olmesartan (OL) developed to overcome the poor permeability of OL. OLM is an angiotensin receptor blocker and is commonly used to treat hypertension. However, OLM has low water solubility and low bioavailability of 26%. It is understood that OLM is unstable in aqueous media; however, this hydrolysis has not been specifically studied in a way that has produced reliable, publishable data. Previously published analytical methods tend to focus mainly on quantitative measurement of OLM, but not quantitative measurement of OL. The objective of this study was to investigate the solubility and aqueous hydrolysis of OLM in different pH buffers by developing an analytical method for the simultaneous measurement of OLM and OL. A novel HPLC method was developed and validated to simultaneously quantify OLM and OL. The solubility of OLM was pH-dependent 37°C, which could lead to food effects and precipitation of OLM in the small intestine. The aqueous hydrolysis of OLM was rapid and significant and followed the zero-order kinetic model with different hydrolysis rates varying across different pH levels in the order: pH 1.2 < pH 3.5 < pH 4.6 ≈ pH 6. These findings indicate that, in addition to low water solubility, aqueous hydrolysis in the gastrointestinal tract contributes to OLM’s low bioavailability. The study emphasizes the importance of fully understanding the solubility and hydrolysis of ester-based prodrugs. Strategies that protect OLM from hydrolysis could have the potential to enhance its bioavailability. Considering ester prodrugs are a key strategy to improve bioavailability, our study in this manuscript is significant for drug formulation development.

## 1 Introduction

Since its approval in 2002, olmesartan medoxomil ((5-methyl-2-oxo-1,3-dioxol-4-yl) methyl 5-(2-hydroxypropan-2-yl)-2-propyl-3-[[4-[2-(2H-tetrazol-5-yl)phenyl]phenyl]methyl] imidazole-4-carboxylate) (OLM) has become a popular, cost-effective choice for the treatment of hypertension. Studies have noted its superiority in producing short- and long-term blood pressure control in general populations compared to other, older drugs in the angiotensin receptor blocker class [1, 2]. Interestingly, it has been studied as having potential applications in the realm of cancer treatment, particularly for pancreatic cancer and lung cancer [3, 4]. The oral bioavailability of OLM is low about 26% [5, 6]. In attempts to raise this value, some have experimented with new formulations of OLM, making it into intranasal or transdermal delivery forms [7–12]. New prodrug forms of OLM have even been made and studied [13, 14]. The reasons for low bioavailability are unclear, but it is likely due to low aqueous solubility, efflux transportation, and unfavorable hydrolysis in the gastrointestinal (GI) tract [5, 6].

OLM is an ester prodrug of olmesartan (OL). The pKa of OL is 4.3, and the log D is -1.2 at pH 7 [15], leading to low permeability through cell membranes. Thus, the prodrug approach was used to convert OL to OLM to increase permeability. However, this prodrug strategy increases the lipophilicity (log P 4.7), leading to low water solubility of OLM [9]. Under the Biopharmaceutics Classification System (BCS), OLM is listed as a BCS Class II drug, which means that it is poorly soluble but highly permeable [16]. The solubility of OLM at different pHs has not been fully studied although the solubility in dissolution media in the present and absence of surfactants has been reported [17]. In addition, OLM is supposed to be stable and absorbed in the GI tract and then enters the blood circulation where OLM is converted to OL by enzymes to provide the therapeutical effect. Ester prodrugs can enhance oral bioavailability only if they remain stable within the GI tract. However, OLM has the potential to hydrolyze to OL in the GI tract by water and enzymes. There is no publication about the hydrolysis kinetics of OLM in aqueous solutions. A deep understanding of OLM aqueous hydrolysis will facilitate the development of new OLM formulations to increase bioavailability.

Because both forms of OLM and OL are present in the GI tract after oral administration of OLM, it is vital to quantitatively measure OLM and OL at the same time during formulation development and evaluation. Various methods have been developed to measure OLM such as HPLC [18–31], UPLC [32], HPTLC [24,33,34], UV-Vis, and other forms of spectrophotometry [17,24,35–42], or even fluorescence spectroscopy [43]. Among them, HPLC methods are reliable and sensitive to quantitatively measure OLM concentrations. However, previously published HPLC methods focused on the measurement of OLM for dissolution or stability in dosage forms, bulk drugs, and drug substances (e.g., tablets). In these studies, the force degradation (e.g., in 0.1 HCl at 60°C for 60 min) was conducted to generate degradation products for HPLC method development, and OL was detected as a degradation product but not measured quantitatively [18,19,44–46]. There are no quantitative methods for OL as well as quantification of the hydrolysis of OLM to OL under physiological temperature (i.e., 37°C) for pre-formulation and formulation development.

The objective of this study was to investigate the solubility and aqueous hydrolysis of OLM in different pH buffers by developing an analytical method for the simultaneous measurement of OLM and OL. We successfully developed and validated a novel HPLC method that accurately analyzes the hydrolysis and solubility characteristics of OLM by quantifying both OLM and its hydrolyzed product OL under various buffer systems at physiological conditions. To the best of our knowledge, this is the first report describing a simultaneous HPLC method for the quantitative measurement of OLM and OL, as well as for assessing OLM’s aqueous solubility and hydrolysis across a range of pH levels.

## 2 Materials and methods

### 2.1. Materials

OLM powder was purchased from TCI American (Portland, OR), and OL powder was purchased from Santa Cruz Biotechnology (Dallas, TX). Acetonitrile and methanol were purchased from Fisher Scientific (Pittsburgh, PA). HPLC-grade phosphoric acid (H_3_PO_4_) was purchased from Sigma-Aldrich (St. Louis, MO). Syringe filters of 0.22 μm pore size were purchased from Celltreat Scientific Products (Pepperell, MA).

### 2.2 HPLC Method Development

#### 2.2.1 Sample preparation.

In developing the HPLC method used for this study, separate stock solutions of OLM 100 μg/mL in acetonitrile and OL 100 μg/mL in methanol were prepared. From here, standard solutions (1, 5, 10, 25, and 50 μg/mL) of either OLM or OL were freshly prepared by serial dilution using mobile phase (methanol: 0.1% H_3_PO_4_ in water at a 1:1 ratio, v/v) right before each measurement. To prepare the combination of OL/OLM standards, an equal amount of OL 50 μg/mL and OLM 50 μg/mL solutions was mixed and diluted with mobile phase to obtain the first combination standard of 25 μg/mL. Then, a serial dilution was conducted by using the mobile phase to obtain the combinations of 0.05, 0.1, 0.5, 1, 5, and 10 μg/mL. Quality control (QC) samples for testing were prepared by spiking the OL/OLM 25 μg/mL standard solution into simulated gastric fluid (SGF) of pH 1.2 and simulated intestinal fluid (SIF) of pH 6. The QC samples included concentrations of 3.1 μg/mL, 6.3 μg/mL, and 12.5 μg/mL. Testing buffers were prepared according to a previous publication [47].

#### 2.2.2 HPLC method.

The HPLC method was developed, optimized, and validated using a Waters ARC HPLC System, equipped with a Quaternary Solvent Manager-R, FTN-R Sample Manager, and a 2489 UV/VIS detector (Milford, MA, USA). OL and OLM were detected at a wavelength of 243 nm. The flow rate was 1.0 mL/min. An Eclipse XDB C18 column (3.5 μm, 4.6 x 100 mm, 4.5 μm particle size; Agilent, USA) was used. For the mobile phase, solvent A was pure methanol, and solvent B was 0.1% H_3_PO_4_ in water. The injection volume was 20 μL. Isocratic elution and gradient elution were tested to optimize peak separation. After optimization, the gradient elution was chosen, in which the solvent A was set at 50% for 2 min, changed to 60% at 4 min and back to 50% at 6.1 min, and then maintained at 50% to 8.5 min. Therefore, a mixture of solvent A and solvent B at a 1:1 ratio (v/v) was used as a mobile phase to prepare samples in the study.

### 2.3. HPLC method validation

The HPLC method developed for this study was validated according to ICH guidelines Q2(R1) [48]. It was validated on the parameters of specificity, linearity, accuracy, precision, range, limit of detection (LOD), limit of quantitation (LOQ), and robustness.

#### 2.3.1 Specificity.

Specificity was tested by comparing the elution peaks produced from testing the mobile phase alone to those produced from testing the OL/OLM standards. Thus, the mobile phase was a blank sample created to ensure that peaks seen on generated chromatograms could be reliably identified as the pure drug substances. Each experiment was repeated in triplicate.

#### 2.3.2 Linearity.

Linearity was tested over the OL/OLM standard concentrations of 0.05 μg/mL to 25 μg/mL. These samples were each tested in triplicate, and the measured areas under the curve for each drug standard were used to create a graph to determine linearity over the given concentration range. From the graphs, a regression equation and correlation coefficient (R^2^) were found; an R^2^ value of 0.999 or better was used for this study. Each experiment was repeated in triplicate.

#### 2.3.3 Accuracy.

Accuracy was determined using the QC samples made with SGF and SIF as described in Section 2.2.1. These QC samples were tested in replicates (n=6) over three days and used to calculate coefficient of variation (CV) values and percent recovery. Accuracy was calculated as (the mean measured concentration)/(the nominal concentration) x 100%.

#### 2.3.4 Precision.

Both intra- and inter-day precisions were tested using the OL/OLM QC samples as described in Section 2.2.1. Intra-day precision was determined by testing each QC sample six times in one day, while inter-day precision was determined by testing the QC samples over three days. Both intra- and inter-day precisions (n=6) were evaluated based on CV values. Precision was expressed as the CV% calculated as (the standard deviation of measured concentrations)/(mean of measured concentrations) x 100%.

#### 2.3.5 Range.

Range was evaluated by examining data used to validate the parameters of linearity, accuracy, and precision, and confirming that all were within an acceptable range as defined by the ICH Q2(R1) guidelines [48].

#### 2.3.6 Limit of detection and limit of quantitation.

The LOD for this study was found by diluting the lowest concentration of OL/OLM standard, 0.05 μg/mL, even further until the signal-to-noise ratio for both the OL and OLM components of the mixture was approximately 3.3. The LOQ was determined by examining the standard curves produced by testing the OL/OLM standards over its entire concentration range in triplicate. It was found by determining the lowest concentration of standard that still produced a reasonable CV% (<2%) for each component present.

#### 2.3.7 Robustness.

The robustness of this study was tested using the OL/OLM QC samples as described in Section 2.2.1. As these samples were prepared in SGF and SIF, the validation of this parameter was conducted by calculating the recovery rate of the samples subjected to these buffers and pH values. Each experiment was repeated in triplicate.

### 2.4 Measurement of OLM solubility in different buffers

To simulate an in vivo condition, a heat block with gentle mixing was set to 37°C, and its temperature was periodically monitored using a thermometer. The buffer at pH 1.2 was prepared by using HCl, and the buffers at 3.5, 4.6, and 6 were prepared by using potassium dihydrogen phosphate adjusted by 1M NaOH or 1M HCl to the determined pH. OLM powder (~ 1 mg) was poured into each buffer (15 mL) and gently mixed at 37°C. At 1, 2, 3, and 28 hours, 3 mL of each sample were withdrawn, 2 mL of the sample was initially passed through a 0.22 μm filter and discarded, and then 1 mL filtrate was collected and diluted with the mobile phase at a 1:1 ratio for HPLC measurement. Each experiment was repeated in triplicate.

### 2.5 Evaluation of OLM hydrolysis kinetics in different buffers

Three kinetic models including zero-, first- and second-order kinetic models were investigated and examined from the linear regression plotting using the equations below, respectively:


$${\text{Zero-order kinetics: C}}_{\text{t}}{\text{= C}}_{\text{0}}{\text{– k}}_{\text{0}}\text{t}$$ (1)

$$\text{First-order kinetics: ln}\left({\text{C}}_{\text{t}}\right)\text{= ln}\left({\text{C}}_{\text{0}}\right){\text{– k}}_{\text{1}}\text{t}$$ (2)

$${\text{Second-order kinetics: 1/C}}_{\text{t}}{\text{= 1/C}}_{\text{0}}{\text{+ k}}_{\text{2}}\text{t}$$ (3)

C_0_ is the OLM concentration at 1 hour, C_t_ is the OLM concentration at time t, and k_0_, k_1_, and k_2_ are the kinetic rates of the zero-, first-, and second-order models, respectively. The solubility data at 1 hour, 2 hours, 3 hours, and 28 hours from Section 2.4 was used for the kinetic modeling. The percentage of OLM at each time point was calculated as %OLM = (OLM mole/mL)/(OLM mole/mL + OL mole/mL) x 100%. OLM concentration or %OLM vs time was fitted by a linear regression for each kinetic model. Residuals were calculated by differences between the observed and predicted OLM concentration at each time point. Residual analysis was performed by plotting residuals vs. predicted values for each model. The R^2^ values and residual analysis were combined to evaluate the good fitting and determine the correct kinetic model.

### 2.5 Statistical analysis

Data are reported in terms of mean ± standard deviation (SD) (n=3 or n=6). CV% is important in determining the statistical legitimacy of generated data and is reported as ((SD)/mean) x100%.

## 3 Results

### 3.1 HPLC method development

**Fig 1:**
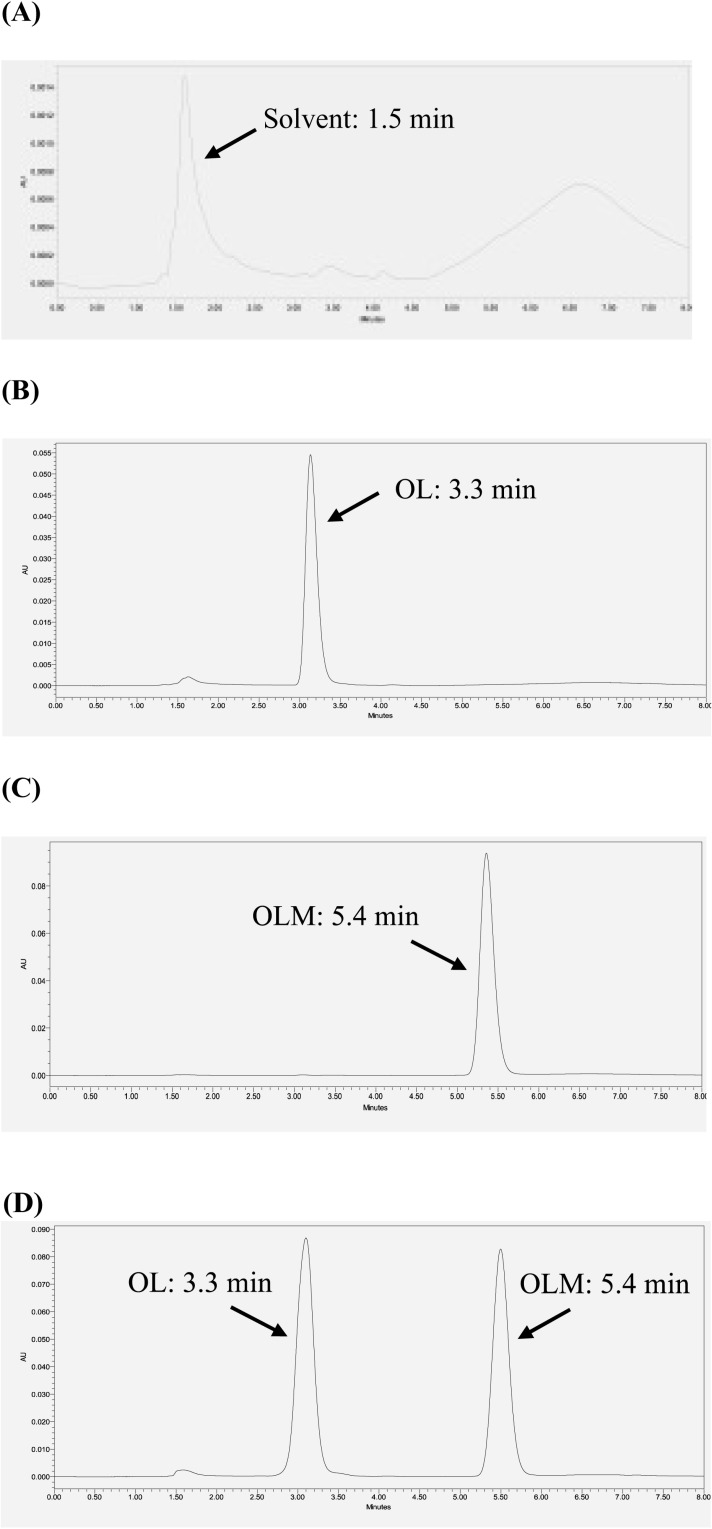
Chromatograms of blank solvent, OL standard solution, OLM standard solution, and a mixture of OLM and OL standards. (A) depicts the mobile phase used for the study (50:50 methanol and 0.1% H_3_PO_4_ in Mill-Q water). (B) depicts unique OL peak produced by testing of OL 25 μg/mL standard. (C) depicts the unique OLM peak produced by testing the OLM 25 μg/mL standard. (D) depicts both OL and OLM peaks produced by testing the OL/OLM 25 μg/mL standard mixture.

Various parameters, such as mobile phase composition, pH, sample preparation, and flow rate were optimized to attain the best chromatograph results for both OL and OLM. When OLM standards were prepared in methanol, OLM peaks decreased over time even when the standards were stored at -20°C, indicating the instability of OLM in methanol that could be caused by hydrolysis. When OLM standards were prepared in acetonitrile, OLM peaks did not show up correctly in good shape. However, OLM stock prepared in acetonitrile was stable for at least 6 months at -20°C. Thus, OLM stock was prepared in acetonitrile and OLM standards were freshly prepared by diluting the stock with mobile phase (methanol: 0.1% H_3_PO_4_ in water at a 1:1 ratio, v/v) before each measurement. The pH and the acid used to prepare the mobile phase significantly influenced peak separation and consistency of retention time. Compared with acetic acid at pH 4.5, 0.1% H_3_PO_4_ at pH 2.5 provided the best peak shape and consistent retention time. Thus, the final mobile phase was composed of methanol (solvent A) and 0.1% H_3_PO_4_ in water (solvent B). In addition, isocratic elution cannot separate the peak of the impurities in OL standards from the OL peak. Several gradient elution methods were evaluated. Finally, the optimized gradient elution was determined, which started from 50% solvent A for 2 min, increased to 60% at 4 min and back to 50% at 6.1 min, and kept at 50% to 8.5 min. OL, OLM, and their impurities were completely separated (Fig 1) without carryover. The retention times of OL and OLM were 3.3 min and 5.4 min, respectively.

### 3.2 HPLC method validation

The HPLC method developed for this study was validated according to the parameters of specificity, linearity, accuracy, precision, range, LOD, LOQ, and robustness.

#### 3.2.1 Specificity.

By comparing chromatograms of the mobile phase and the tested OL/OLM standards of concentrations from 0.05 μg/mL to 25 ug/ml, it was determined that the elution peaks of OL and OLM examined were pure, demonstrating the specificity of the optimized method to simultaneously measure OL and OLM.

#### 3.2.2 Linearity and range.

**Fig 2:**
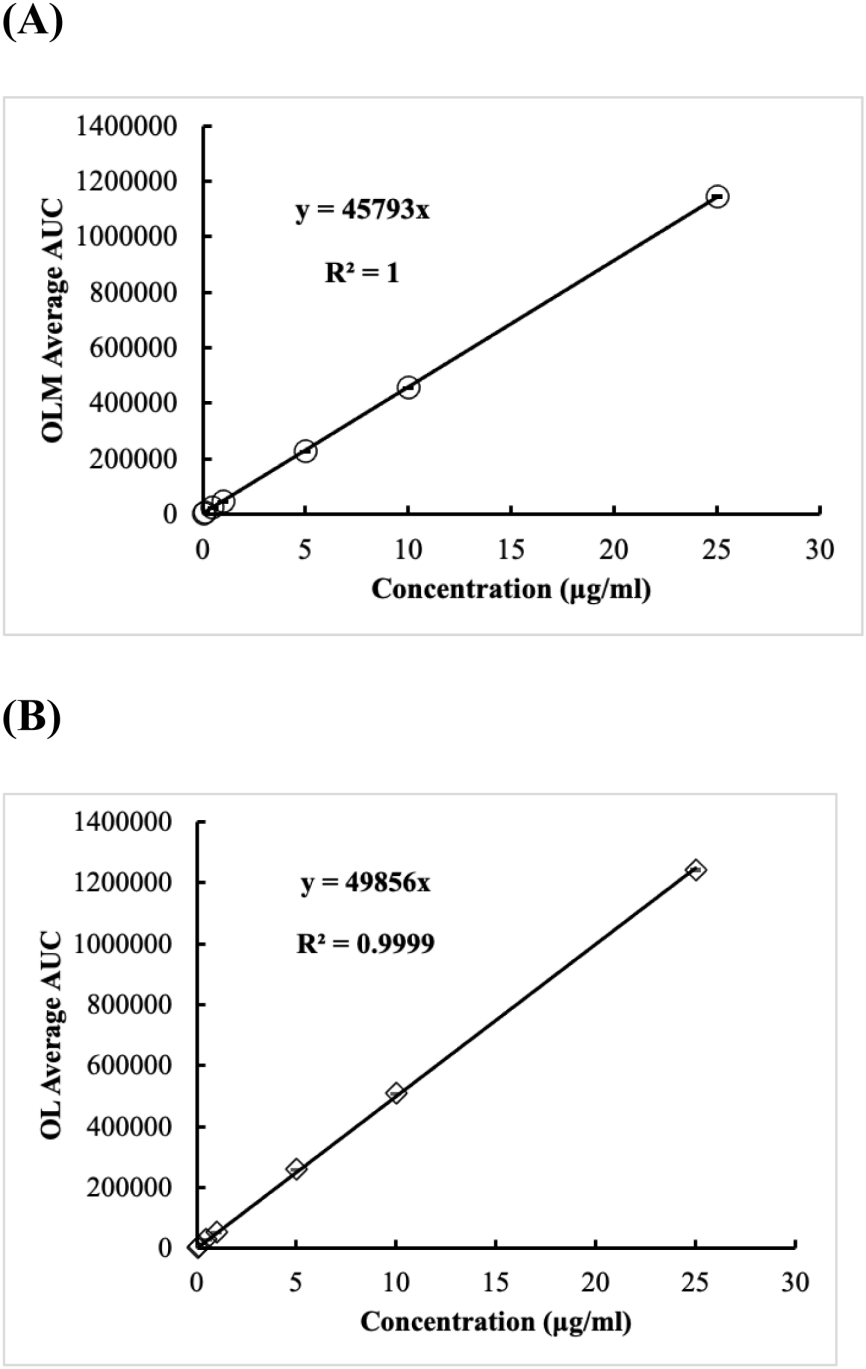
Standard curves generated from the measurement of OL and OLM present in OL/OLM standard mixtures ranging in concentration from 0.05 μg/ml to 25 μg/mL. (A) depicts the curve of OLM. (B) depicts the curve of OL. These figures validate the linearity of analytical method validation under current ICH guidelines.

After testing the OL/OLM standards of concentrations from 0.05 μg/mL to 25 μg/mL, both OL and OLM showed linear responses. For OLM, as shown in Fig 2A, the regression equation was Y=45793X, and the R^2^ value was 1. For OL, as shown in Fig 2B, the regression equation was Y=49856X, and the R^2^ value was 0.9999.

#### 3.2.3 Accuracy and robustness.

The data for accuracy are shown in Tables 1 and 2. At the three concentrations of the SGF- and SIF-based QC samples tested (3.1 μg/mL, 6.3 μg/mL, and 12.5 μg/mL), the CV value was less than 3% for both OL and OLM. The recovery rates for OL and OLM across each concentration and each condition fell within the accepted range of 90–110% of the expected value. Thus, the HPLC method was accurate and robust.

**Table 1:** Intra- and inter-day precision and accuracy for OLM in QC samples.

QC Buffer	Conc. (μg/ml)	Accuracy and precision of OLM in SGF and SIF QC samples				
Day	Day 1 (n=6)	Day 2 (n=6)	Day 3 (n=6)	Overall (n=18)
		Mean	3.15	3.16	3.28	3.20
SGF	3.1	SD	0.009	0.003	0.004	0.063
		CV (%)	0.3	0.09	0.11	1.96
		Recovery (%)	100.8	101.2	105.1	102.3
		Mean	6.22	6.43	6.5	6.38
	6.3	SD	0.01	0.009	0.014	0.12
		CV (%)	0.16	0.14	0.21	1.89
		Recovery (%)	99.6	102.8	103.9	102.1
		Mean	12.52	12.83	13.14	12.83
	12.5	SD	0.184	0.018	0.022	0.28
		CV (%)	1.47	0.14	0.17	2.18
		Recovery (%)	100.2	102.6	105.1	102.6
		Mean	2.93	2.89	2.92	2.91
SIF	3.1	SD	0.015	0.017	0.068	0.042
		CV (%)	0.52	0.60	2.34	1.45
		Recovery (%)	93.8	92.5	93.3	93.2
		Mean	6.31	6.33	6.42	6.35
	6.3	SD	0.003	0.009	0.011	0.047
		CV (%)	0.04	0.13	0.18	0.74
		Recovery (%)	101.0	101.3	102.6	101.**7**
		Mean	12.93	12.81	13.16	12.97
	12.5	SD	0.024	0.124	0.058	0.170
		CV%	0.18	0.97	0.44	1.31
		Recovery (%)	103.5	102.4	105.3	103.7

a CV(%) = (SD/Mean) x 100%

^b^Recovery(%) = (Measured concentration/theoretical concentration) x 100%

**Table 2:** Intra- and inter-day precision and accuracy for OL in QC samples.

QC Buffer	Conc. (μg/ml)	Accuracy and precision of OL in SGF and SIF QC samples				
Day	Day 1 (n=6)	Day 2 (n=6)	Day 3 (n=6)	Overall (n=18)
		Mean	3.15	3.16	3.28	3.20
SGF	3.1	SD	0.009	0.003	0.004	0.063
		CV (%)	0.3	0.09	0.11	1.96
		Recovery (%)	100.8	101.2	105.1	102.3
		Mean	6.22	6.43	6.5	6.38
	6.3	SD	0.01	0.009	0.014	0.12
		CV (%)	0.16	0.14	0.21	1.89
		Recovery (%)	99.6	102.8	103.9	102.1
		Mean	12.52	12.83	13.14	12.83
	12.5	SD	0.184	0.018	0.022	0.28
		CV (%)	1.47	0.14	0.17	2.18
		Recovery (%)	100.2	102.6	105.1	102.6
		Mean	2.93	2.89	2.92	2.91
SIF	3.1	SD	0.015	0.017	0.068	0.042
		CV (%)	0.52	0.60	2.34	1.45
		Recovery (%)	93.8	92.5	93.3	93.2
		Mean	6.31	6.33	6.42	6.35
	6.3	SD	0.003	0.009	0.011	0.047
		CV (%)	0.04	0.13	0.18	0.74
		Recovery (%)	101.0	101.3	102.6	101.**7**
		Mean	12.93	12.81	13.16	12.97
	12.5	SD	0.024	0.124	0.058	0.170
		CV%	0.18	0.97	0.44	1.31
		Recovery (%)	103.5	102.4	105.3	103.7

a CV(%) = (SD/Mean) x 100%

^b^Recovery(%) = (Measured concentration/theoretical concentration) x 100%

#### 3.2.4 Precision.

The data for precision are shown in Tables 1 and 2. As the CV was less than 3% for each condition and across the testing interval of three days, both intra- and inter-day precision were validated for this HPLC method.

#### 3.2.5 Limit of detection and limit of quantification.

Through further dilution of the 0.05 μg/mL OL/OLM standard, the LOD for both OL and OLM was found to be 0.03 μg/mL. The LOQ was determined by examining the data produced by testing the OL/OLM standards across the concentration range of 0.05 μg/mL to 25 μg/mL. The LOQ was 0.1 μg/mL, as this was the lowest concentration standard that produced an acceptable CV% value (<2%).

### 3.3 Solubility of OLM in different pH buffers

**Fig 3:**
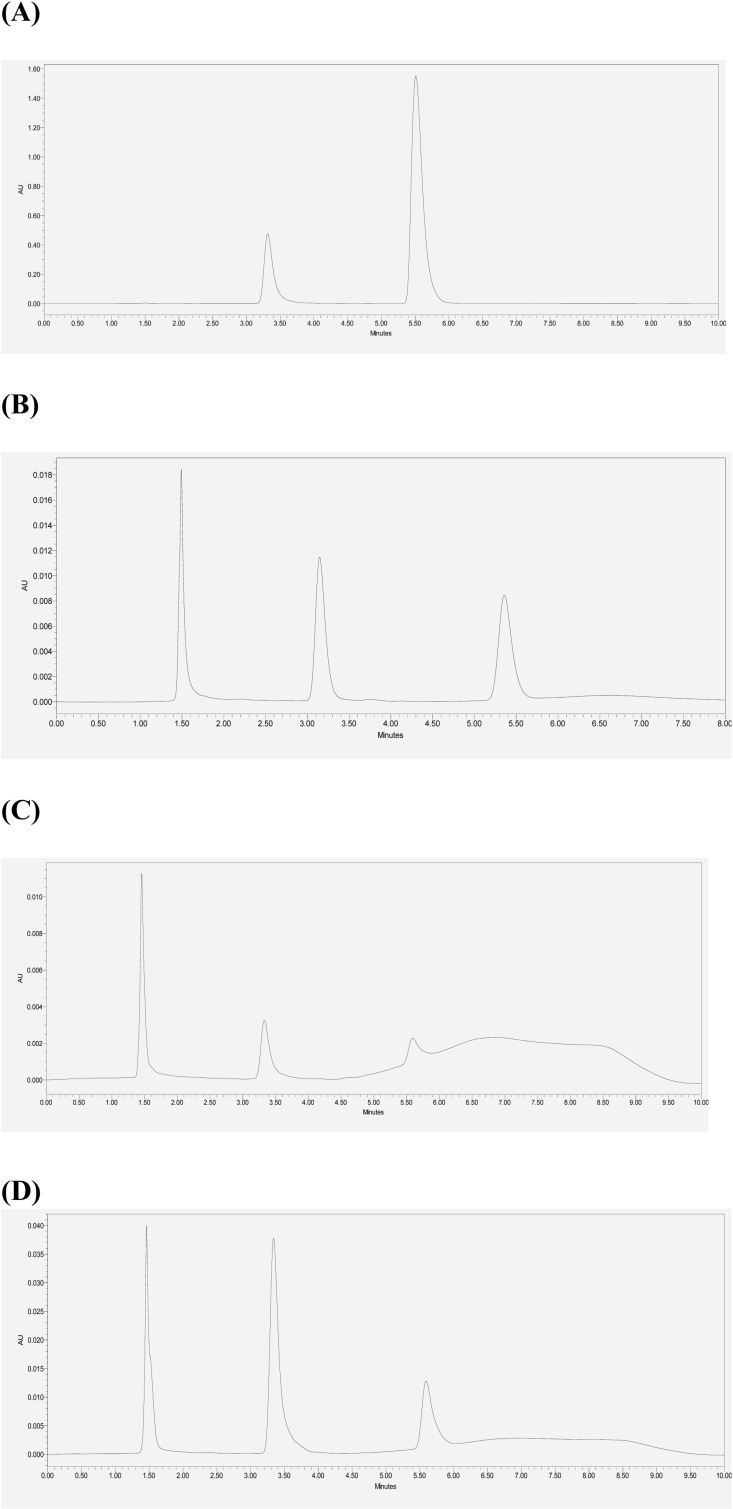
Chromatograms of OLM solubility samples in different pH buffers. OLM and OL were simultaneously detected in the solubility samples, demonstrating the degradation of OLM. Chromatograms were produced from solubility testing of OLM (A) in pH 1.2 buffer, (B) in pH 3.5 buffer, (C) in pH 4.6 buffer, and (D) in pH 6 buffer at 1 hour. Peak identification: (1) peak at 1.5 min is the solvent peak, (2) peak at 3.3 min is the OL peak and (3) peak around 5.5 min is the OLM peak.

**Fig 4:**
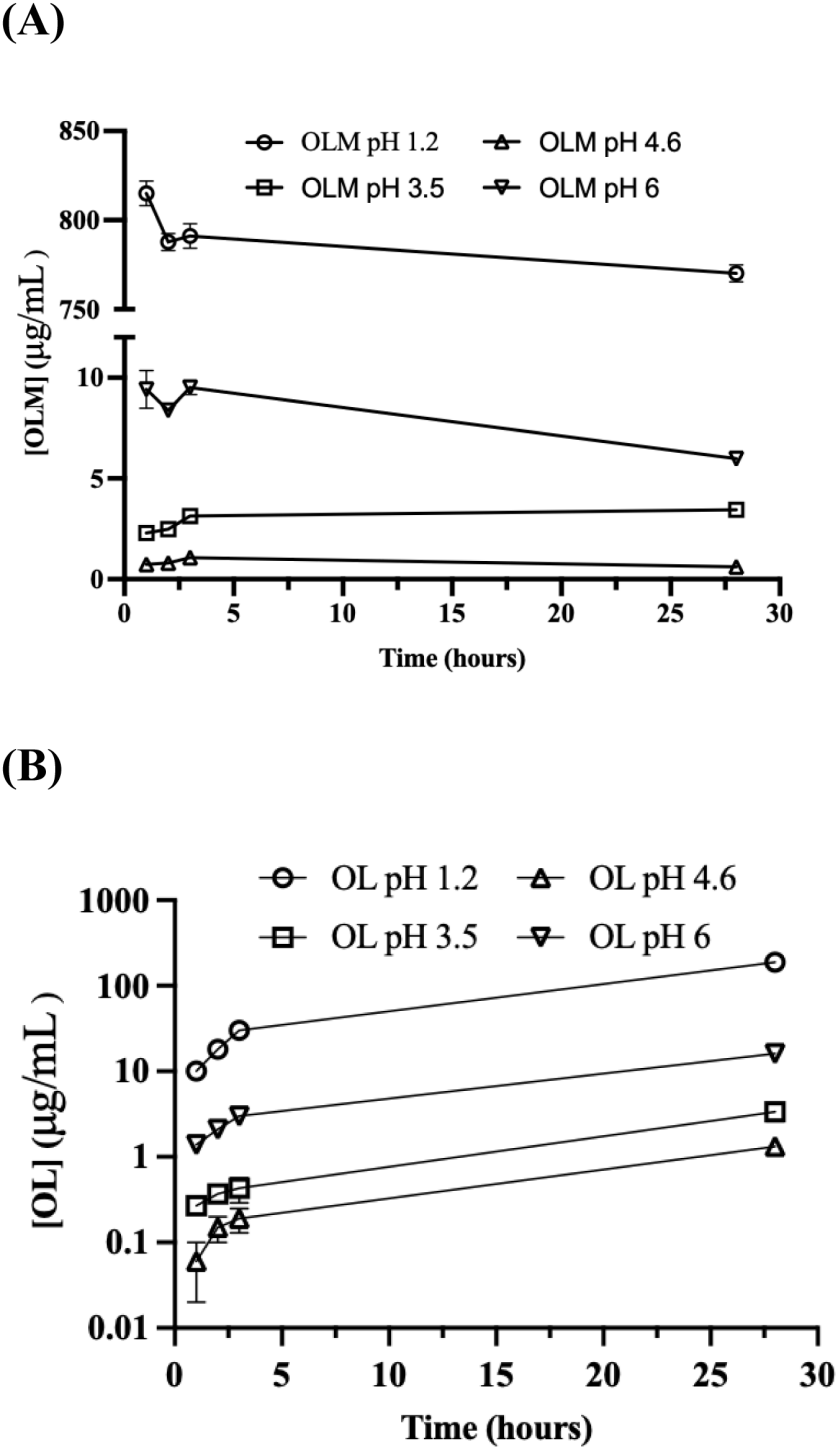
Solubility measurements of OLM in different pH buffers at 1, 2, 3, and 28 hours. (A) the concentrations of OLM in each buffer over 28 hours. (B) the corresponding concentrations of OL in each buffer over 28 hours.

**Fig 5:**
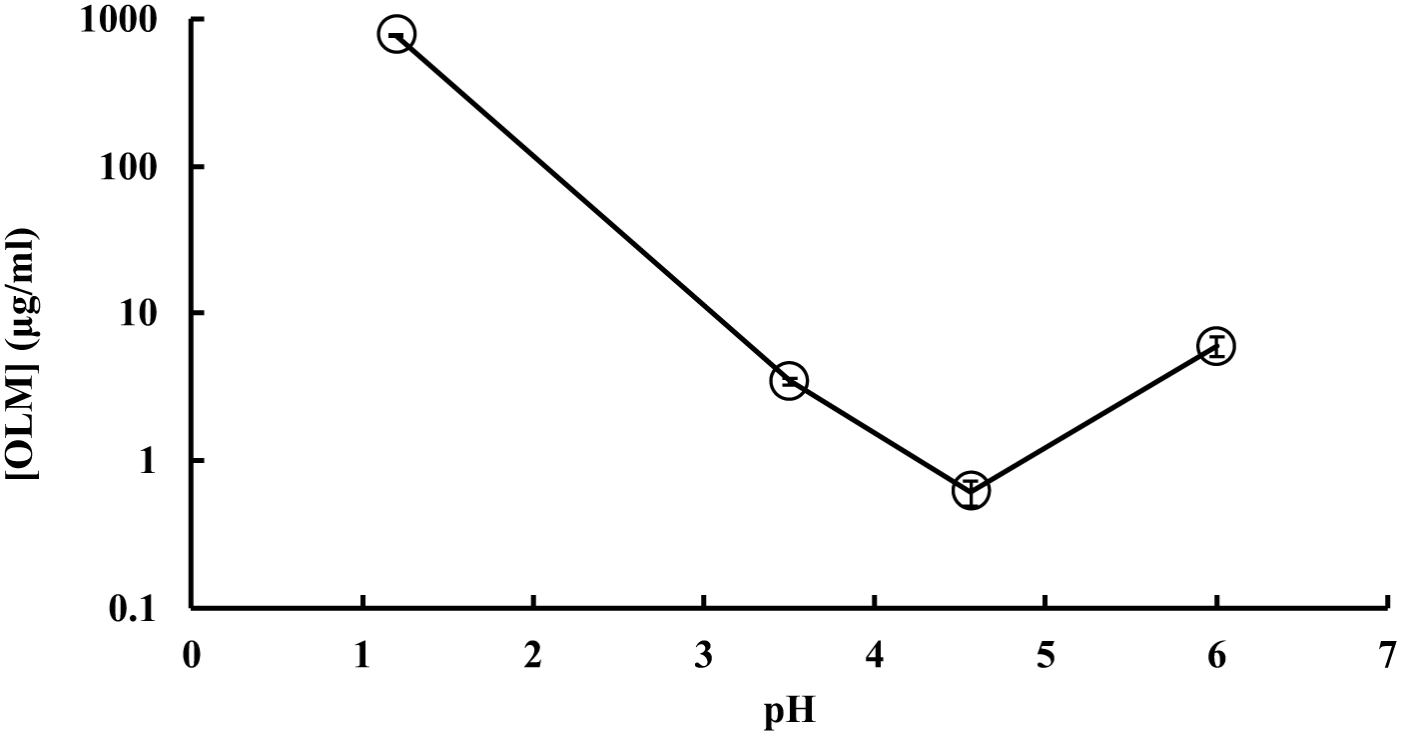
Saturated solubility of OLM at different pHs measured at 28 hours.

The solubility of OLM was measured in pH 1.2, 3.5, 4.6, and 6 buffers at 37°C at 1 hour, 2 hours, 3 hours, and 28 hours, and, at the same time, the hydrolysis product OL was quantitatively measured by the HPLC method. The peak of OL was detected in the OLM solubility samples in each buffer starting at 1 hour, indicating the degradation of OLM (Fig 3). The concentrations of OLM in pH 1.2 and 6 buffers decreased while those in pH 3.5 and 4.6 buffers increased over time (Fig 4A). As shown in Fig 4B, OL concentration increased in each buffer over time, confirming the hydrolysis of OLM. Besides OL, no other degradation products were detected in the samples. The solubility of OLM in the tested buffers was pH-dependent as following the order: pH 1.2 > pH 6 > pH 3.5 > pH 4.6. After 2 hours, solubilization of OLM in each buffer was close to equilibration and the OLM concentrations at 28 hours can be considered as the saturated solubility. Thus, the pH-dependent solubility of OLM is shown in Fig 5 by using the data at 28 hours.

### 3.4 Kinetic modeling of OLM aqueous hydrolysis in different pH buffers

**Fig 6:**
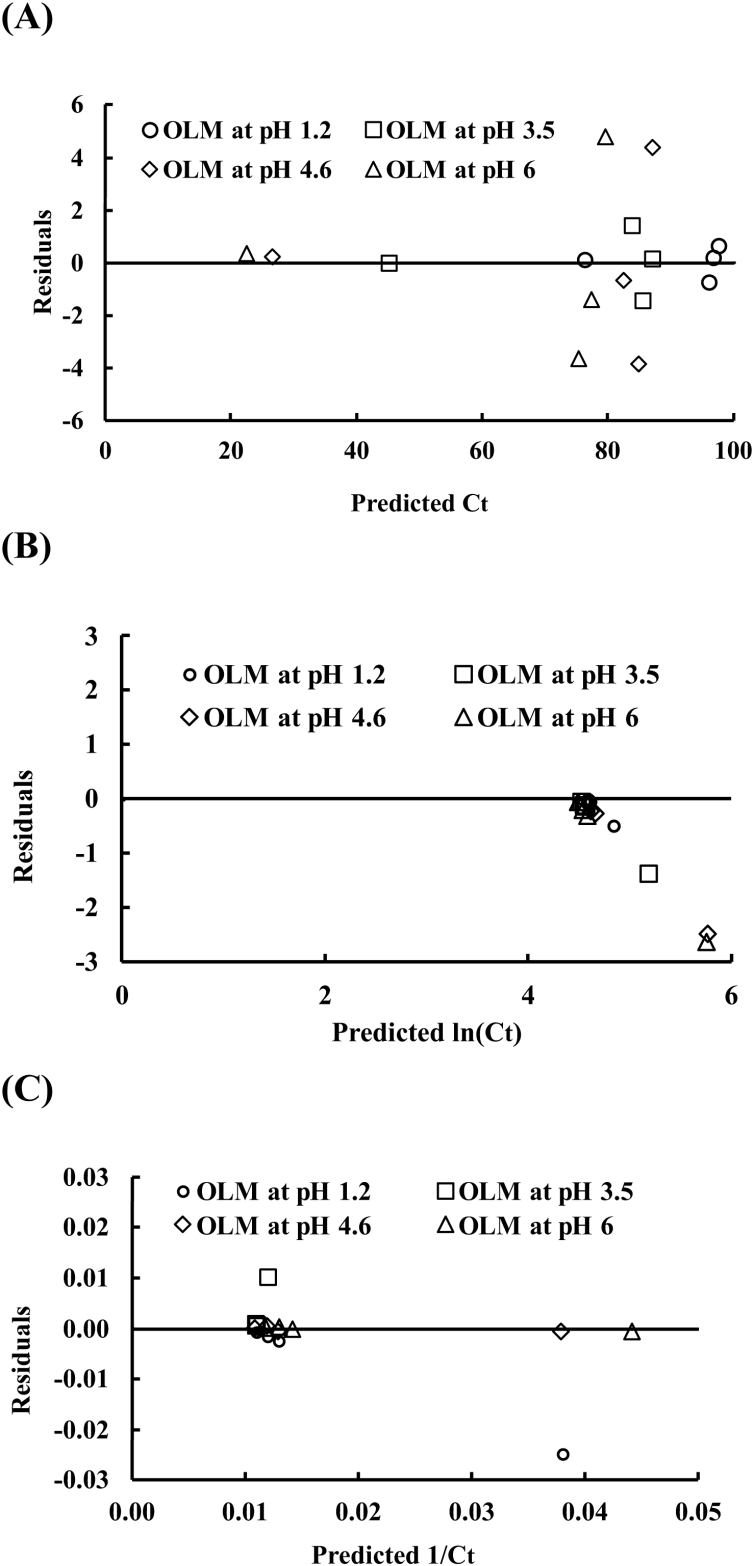
Residual analysis of the linear regression plotting of OLM hydrolysis data with the zero-order kinetic model (A), first-order kinetic model (B), and second-order kinetic model (C).

**Fig 7:**
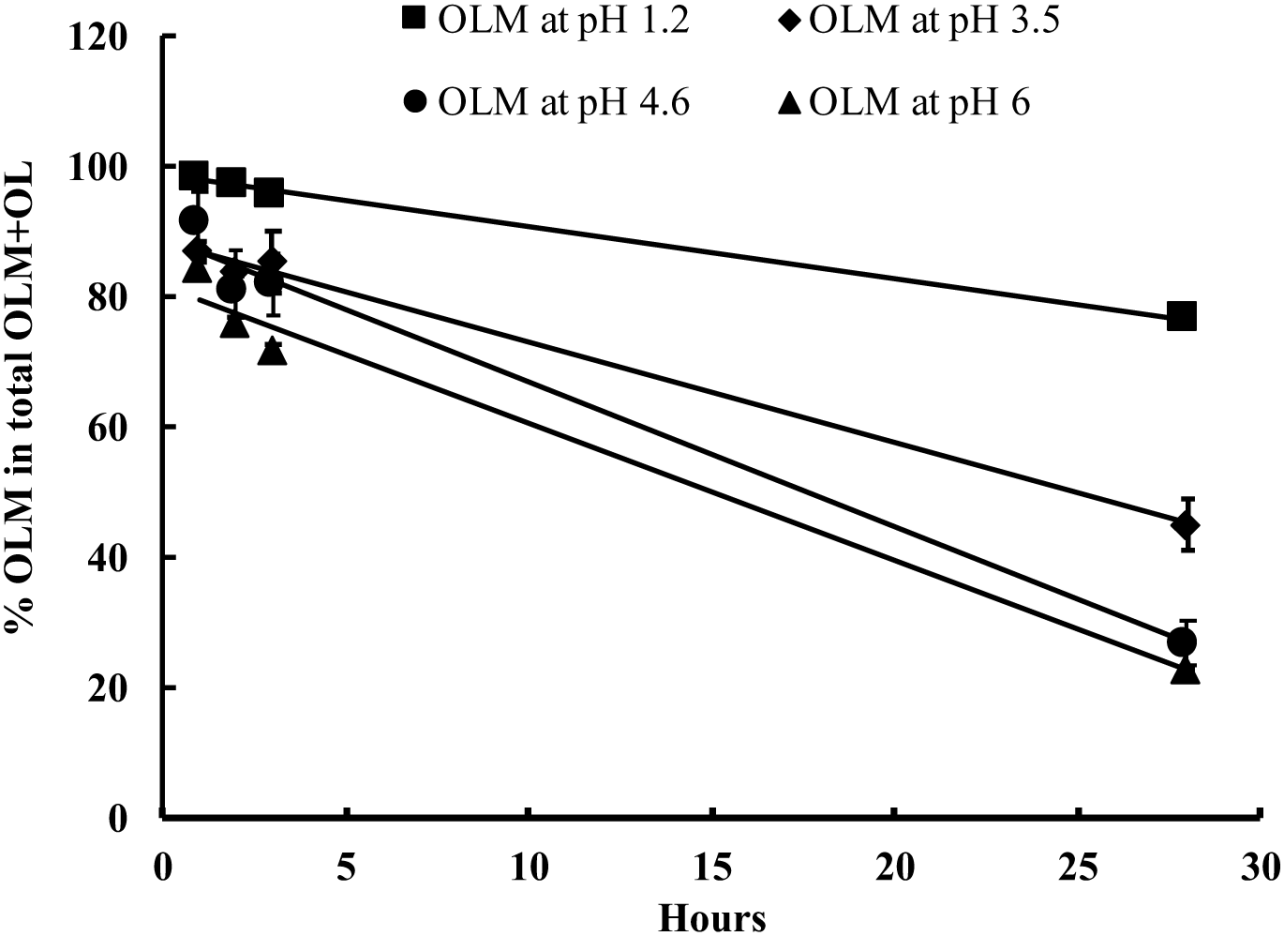
Fitting of OLM hydrolysis in different pH buffers at the zero-order kinetic model.

Initially, OLM concentration was used to generate the plots using Equations (1), (2), and (3) for zero-, first- and second-order kinetic model, respectively. All plots were not linear, e.g., R^2^=0.333 for a zero-order model, which was explicable because the process in the solubility measurement included solubilization and equilibration in addition to OLM hydrolysis. Because we quantitatively measured both OLM and OL, we were able to measure OLM hydrolysis kinetics by using %OLM, instead of OLM concentration. The fitting using %OLM showed linearity. However, all fittings had R^2^ values between 0.986–0.999 no matter if using zero-, first- or second-order equation. Thus, a further evaluation using residual analysis was conducted by plotting residuals against predicted values. As shown in Fig 6, only the residual plot of the zero-order model shows random distribution without discernible patterns, demonstrating the zero-order model is the best fit. The fitting of the OLM hydrolysis data with the zero-order model is shown in Fig 7 and the parameters are shown in Table 3.

**Table 3:** Parameters of the zero-order kinetic model of OLM hydrolysis to OL in different pH buffers.

Buffers	Rate equation	R^2^ for fitting	Rate constant	Reaction order
**1.2**	y=98.6-0.789x	0.9967	0.789	Zero-order
**3.5**	y=88.7-1.55x	0.9966	1.55	Zero-order
**4.6**	y=89.4-2.24x	0.9866	2.24	Zero-order
**6**	y=81.8-2.11x	0.9834	2.11	Zero-order

As shown in Fig 7 and Table 3, the aqueous hydrolysis of OLM was rapid and significant and followed the zero-order kinetic model with different hydrolysis rates varying across different pH levels in the order: pH 1.2 < pH 3.5 < pH 4.6 ≈ pH 6. Over 28 hours, 18% of dissolved OLM was hydrolyzed to OL in pH 1.2 buffer, 41% in pH 3.5 buffer, 61% in pH 4.6 buffer, and 60% in pH 6 buffer. Moreover, the hydrolysis of OLM in pH 6 buffer was pronounced, showing within 1 hour 13% OLM were converted to OL.

## 4 Discussion

OLM as an ester is known for its potential for aqueous hydrolysis, but no publications have reported the OLM solubility and hydrolysis in different buffers. After hydrolysis, OLM produces OL in water. Thus, a validated and reliable analytical method to simultaneously quantify OLM and OL is mandatory to evaluate OLM solubility and hydrolysis. OLM has been measured by various analytical methods, but there is no report on quantitatively measuring OL. In the present study, we successfully developed and validated a novel HPLC method to simultaneously measure OLM and OL in SGF (pH 1.2) and SIF (pH 6.7). During HPLC method development, we noticed the instability of OLM in methanol and irregular peak shapes of OLM samples prepared by acetonitrile and methanol. Thus, we carefully investigated the sample preparation approach and used the mobile phase to dilute samples before HPLC measurement to solve the issues. To mimic the aqueous environments in the GI tract, we used SGF and SIF to prepare the QC samples for HPLC method validation. The data shows that the HPLC method is precise and accurate (Table 1 and Table 2). Because we can simultaneously quantify both OLM and OL in solubility samples, we were able to use %OLM to conduct kinetic modeling for OLM hydrolysis (Fig 7). The OLM hydrolysis followed the zero-order kinetic model, meaning that the OLM hydrolysis is concentration-independent.

Most published formulation strategies such as micelles, solid lipid nanoparticles, and self-microemulsion focused on overcoming low OLM solubility. However, the solubility of OLM was dependent on pH (Fig 4 and Fig 5). Considering the dose of OLM is 20 mg once a day, OLM has sufficient solubility (815 μg/mL) to dissolve in the fasting stomach (pH 1.4–2.1), whereas it is insoluble (1–3 μg/mL) in the fed stomach (pH 3–7). Although it is currently recommended to take OLM orally with or without food, our data indicates that food and/or stomach pH might influence OLM absorption. The solubility of OLM at pH 1.2 was over 80-fold higher than that at pH 6 (~ 9 μg/mL). If dissolved OLM in the fasting stomach (pH 1.2) is not absorbed rapidly in the small intestine (pH 6–7), it could precipitate in the small intestine, which would cause low bioavailability. In addition, OLM hydrolyzed to OL starting at 1 hour in each buffer (Fig 3 and Fig 4). Thus, dissolved OLM can be converted to OL in the stomach and the small intestine and lose its permeability, leading to low absorption. Currently, there is no report on formulation strategies that could protect OLM from water and enzyme hydrolysis, likely because of the lack of emphasis and understanding of OLM’s pH-dependent solubility and hydrolysis. In addition to solubility, one needs to consider the potential hydrolysis and precipitation of OLM in the GI tract to improve bioavailability. Nanoparticles [49] and cyclodextrins [50] have been reported to protect drugs against hydrolysis. Precipitation inhibitors such as HPMC and PVP [51] could be added to prevent OLM precipitation in the small intestine caused by the solubility decrease from pH 1.2 to 6.8. Moreover, local targeting delivery systems such as pH-responsive formulations and enteric-coated formulations [52] could be used to release OLM in the small intestine to reduce hydrolysis in the GI tract.

This present study also emphasizes the importance of simultaneous measurement of ester prodrug and parent drug during drug formulation characterization and development. Ester-based prodrugs have been widely used to improve solubility and/or permeability. However, one should keep in mind that ester-based prodrugs have the potential for aqueous hydrolysis in the GI fluid, leading to degradation and low bioavailability. In addition, in vitro cell uptake and permeability studies have been used to evaluate and optimize drug formulations. Permeable prodrugs could degrade to non-permeable parent drugs in the media used for these cell studies, which could mislead the data interpretation and overall permeability evaluation. Aqueous hydrolysis also could happen during dissolution studies. Without measuring the parent drug, the dissolution of a prodrug cannot be correctly understood.

## 5 Conclusion

We successfully developed and validated a novel HPLC method to simultaneously quantify OLM and OL. Using this method, we demonstrated that OLM exhibits pH-dependent solubility, which may contribute to food effects and drug precipitation in the small intestine. Additionally, the aqueous hydrolysis of OLM to OL was rapid and followed zero-order kinetics in each buffer, with the effect being more pronounced at pH 6. This could lead to a loss of permeability and reduced absorption of OLM.

Our research primarily focused on the impact of pH and the aqueous environment on OLM hydrolysis. It is important to note, however, that in vivo conditions in the GI tract are far more complex than those in vitro simulations. Factors such as enzymes, bile salts, and proteins also influence OLM hydrolysis and absorption in vivo. Despite these limitations, the study highlights the critical importance of understanding the solubility and aqueous hydrolysis of ester-based prodrugs. Future studies involving more complex and physiologically relevant conditions are warranted. Overall, this study provides a framework for investigating the aqueous hydrolysis of ester-based prodrugs and offers insights into their formulation development.
